# Monthly Summary

**Published:** 1883-05

**Authors:** 


### MONTHLY SUMMARY.

Bone and Brain Disease in Syphilis.—At a meeting of the Pathological Society of London, Mr. Victor Horsley exhibited specimens of Bone and Brain Disease in Syphilis. The organs shown possess no special interest beyond the fact that successful treatment lessens the opportunities of studying syphilitic lesions. In this case the patient was admitted into University College Hospital under Mr. Hill, from the Lock Hospital. Soho, in a very weak state, and suffering from pyæmic abscesses, What history could be attained showed his condition to be pyæmic, following on necrosis of the facial and cranial bones. The specimens show, first, the points of necrosis on the frontal and molar bones, together with the spongy bones of the nose, of which the inferior turbinate was found post-mortem to be a mere sequestrum, and kept in the nasal fossa by tenacious muco-pus. The whole mucous membrane of the pharynx is hyperæmic, and shows a few cicatrices of previous ulceration. The seats of active mischief were excessively foul, the smell of the discharge not being controlled by antiseptics. The frontal bone shows very well the cicatrices of former ulceration and destruction of the outer table. The lungs on both sides showed some cirrhosis of the apices and broncho-pneumonia; the liver, fatty and cirrhotic, presented a depressed scar on its surface penetrating a quarter of an inch into the substance of the organ. Both spleen and kidneys were cirrhotic, while the former was greatly enlarged, being seven inches long by four inches and a half by two inches. The other abdominal organs showed no particular lesion. On removing the brain there was found an excess of cerebro-spinal fluid, while the arachnoid and pia matar at the base were opaque, and in places matted together by exudation. This did not seem to have caused any paralysis of any cranial nerve. There is asymmetry of the cerebellum, the lateral lobe of the left side being deficient on its under surface at the anterior border, flocculus being scarcely represented. This does not seem to be the result of disease. There were eleven abscesses in the connective tissue of the limbs and trunk.—*London Lancet.*

---

A REMARKABLE CASE was recently tried at the Seine Assizes in Paris. The prosecutor was Dr. Evans, the well-known American dentist, of the Avenue de l'Opera, Paris—the same Dr. Evans who, in 1870, afforded a temporary refuge at his residence to the Empress Eugenie when she was flying from the Tuileries. The prisoner was one William Williamson, late clerk and cashier to Dr. Evans, and a British subject. Williamson has been in the doctor's service for the last ten years, during which he has, according to his own admission, appropriated to himself 300,000 francs, and according to his employer, no less than 1,000,000 francs, from the fees paid by Dr. Evans' patients. "How much, then, sir, do you earn per annum, if you have been robbed at such a rate without discovering it for ten years?" demanded the prisoner's counsel. "Your question, replied Dr. Evans, "is hardly fair; but I have no objection to say that my income is large enough to prevent my missing the loss of 100,000 francs a year." On Williamson being detected, he escaped to Belgium; but despite the objections raised on account of his nationality, he was handed to the French authorities under the Extradition Treaty. Williamson has lived on an extravagent scale for years, and there seems to have been no excuse for Dr. Evans remaining so long ignorant of his dishonesty. Taking this view, the jury accompanied their verdict of guilty with extenuating circumstances, and the sentence passed was three years imprisionment only.

---

LONGEVITY OF OCCUPATIONS.—The *Scientific American* states that the longest lived men are merchants, and then in the order named; weavers, shoemakers, carpenters, blacksmiths, laborers, miners, tailors, bakers, butchers, liquor dealers. The mortality among liquor dealers is so great that in good companies they are admitted with great caution and on short policies. The callings of brewer, it is added, typesetter, tinsmith, lithographer and stonecutter are all unfavorable to longevity. These deductions are made from data found in the office of the Registrar-General of Great Britian.—*Medical and Surgical Reporter.*

---

TO DISGUISE THE ODOR OF IODIFORM.—The *Journal de Therapeutique* says that Dr. Yvon effects the abolition of the smell of iodiform by the very simple procedure of incorporating with it a little essence of roses. Half a drop of the essence removes the odor of sixty grammes of iodiform, the compound retaining that of the essence.—*Medical and Surgical Reporter.*

---

DEATH FROM DICHLORIDE OF ETHIDENE.—The *Lancet* January 27, 1883, records the death of a man who was anæsthetized with the above agent. Nitrite of amyl, and artificial respiration for an hour, were useless. The heart was flabby, thin and extensively degenerated; the valves were healthy.

---

SOUTHERN DENTAL ASSOCIATION.—Change of time of meeting. Dr. L. D. Carpenter, President, announces that the time of meeting of this Association has been changed to Tuesday, July 31st, 1883, in Atlanta, Georgia. Dr. D. Hopps, President of the Georgia State Dental Society, also announces that their meeting will be held on Monday, July 30th 1883, at the same place.

The change of time has been made to accommodate many Southern dentists who wish to attend the meetings of the American and other Associations in August.

